# Inflammatory Responses are Sex Specific in Chronic Hypoxic–Ischemic Encephalopathy

**DOI:** 10.1177/0963689718766362

**Published:** 2018-04-25

**Authors:** Abdullah Al Mamun, Haifu Yu, Sharmeen Romana, Fudong Liu

**Affiliations:** 1 Department of Neurology, The University of Texas Health Science Center at Houston, McGovern Medical School, Houston, TX, USA; 2 Department of Neurology, Shanghai Jiaotong University Sixth People’s Hospital South Campus, Shanghai Fengxian District Central Hospital, Shanghai, China

**Keywords:** Hypoxic–ischemic encephalopathy, inflammation, microglia, neurogenesis, Rice–Vanucci model

## Abstract

Neonatal hypoxic–ischemic encephalopathy (HIE) is increasingly recognized as a sexually dimorphic disease. Male infants are not only more vulnerable to ischemic insult; they also suffer more long-term cognitive deficits compared with females with comparable brain damage. The innate immune response plays a fundamental role in mediating acute neonatal HIE injury. However, the mechanism underlying the sex difference in chronic HIE is still elusive. The present study investigated the sex difference in HIE outcomes and inflammatory response in the chronic stage (30 days after HIE). Postnatal day 10 (P10) male and female C57BL/6 pups were subjected to 60-min Rice–Vanucci model (RVM) to induce HIE. Brain atrophy and behavioral deficits were analyzed to measure stroke outcomes at 30 days of HIE. Flow cytometry (FC) was performed to examine central (microglial activation) and peripheral immune responses. Serum levels of cytokines and sex hormones were determined by enzyme-linked immunosorbent assay (ELISA). Neurogenesis was quantified by 5-Bromo-2′-deoxyuridine (BrdU) incorporation with neurons. Results showed males had worse HIE outcomes than females at the endpoint. Female microglia exhibited a more robust anti-inflammatory response that was corresponding to an enhanced expression of CX3C chemokine receptor 1 (CX3CR1) than males. More infiltration of peripheral lymphocytes was seen in male vs. female HIE brains. Cytokine levels of tumor necrosis factor (TNF)-α and interleukin (IL)-10 were more upregulated in males and females respectively than their counterparts. Neurogenesis was more highly induced in females vs. males. No significant difference in circulating hormonal level was found between males and females after HIE. We conclude that a sex dichotomy in pro- and anti-inflammatory response underlies the sex-specific chronic HIE outcomes, and an enhanced neurogenesis in females also contribute to the sex difference.

## Introduction

Perinatal hypoxic–ischemic encephalopathy (HIE) is a major cause of neonatal death and long-term disability. Clinical data showed about 15–20% of HIE infants will die in the postnatal period, and an additional 25% will develop severe and chronic neuropsychological sequelae, including mental retardation, visual motor or visual perceptive dysfunction, increased hyperactivity, cerebral palsy, and epilepsy^1^. Boys are more sensitive to the ischemic insult and have worse outcomes than girls^2^. Experimental studies have recapitulated the sex difference in acute HIE^3^. However, the chronic HIE outcomes reflect long-term sensorimotor/cognitive deficits and have not been well studied. It has important clinical relevance to study the pathophysiological mechanisms underlying the chronic sex differences in HIE.

Cerebral ischemia triggers activation of microglia, the resident immune cells in the brain, which subsequently initiates and perpetuates immune responses^3–6^. Once activated, microglia develop macrophage-like capabilities including phagocytosis, cytokine production, antigen presentation, and the release of matrix metalloproteinases (MMPs) that disrupt the blood–brain barrier (BBB)^7^. As a result, peripheral immune cells infiltrate into the ischemic area contributing to the exacerbation of ischemic injury due to a secondary neuronal damage^8^. Microglial response to stroke starts from the onset of stroke, and lasts throughout the pathological procedure of HIE, with different characteristics in the acute vs. chronic stage^4,5,9^. Like their ‘paralog cell’ macrophages in the peripheral immune compartments, microglia also exhibit either M1 (pro-inflammatory) or M2 (anti-inflammatory) phenotype after activation depending on the depending on the progression of neuroinflammation^6,9–12^, and the M1/M2 phenotypes overlap with each other with one of the two predominating at a certain stage of the disease^9^. M1-like microglia release neurotoxic inflammatory mediators^13^ to cause neuronal damage, delay post-stroke neurogenesis^14^, prevent axon regeneration^15^ and limit the curative effect of thrombolytic therapy^16^. On the contrary, M2 microglia clear ischemic debris and promote tissue repair through anti-inflammatory signaling, primarily at the recovery stage^10^. M1 and M2 microglial activation status can be differentiated by specific activation markers (e.g. major histocompatibility complex (MHC) II for M1; CD206 for M2^4,6^). In basal conditions, the activation of microglia is strictly controlled by endogenous inhibitory signaling (e.g. CX3CL1-CX3CR1 signaling^17,18^). The chemokine CX3CL1 expressed on neurons (also referred to as fractalkine) is a cleavable transmembrane protein that binds with its G-protein coupled receptor CX3CR1 on microglia to keep microglia quiescent^19^. Loss of CX3CL1-CX3CR1 interaction has been shown to be neurotoxic in many disease models including Parkinson’s disease, traumatic brain/spinal cord injury, and stroke^18,20,21^.

Sex differences have been reported in microglial activation and immune responses in adult animal models. Microglia volume was different in female vs. male mice after a 60-min stroke; female sham microglia had constitutively higher expression of the phagocytic marker CD11b than male microglia, and only males exhibited an increase in CD11b immunoreactivity after ischemia^22^. Increased microglial activation induced by high-fat diet was only seen in CX3C chemokine receptor 1 (CX3CR1) knockout (KO) female mice but not in males^19^. Whether sex differences exist in chronic microglial responses to neonatal HIE is not known. In the present study, we examined CX3CR1 signaling in the chronic microglial response to HIE in both male and female pups and explored the possible mechanisms underlying the sex difference in long-term functional damage in HIE.

## Materials and Methods

### Experimental Animals

All animal protocols were approved by the University’s Institutional Animal Care and Use Committee and were performed in accordance with National Institutes of Health and University of Texas Health Science Center at Houston (UTHealth) animal guidelines. Wild-type C57BL/6 postnatal day 10 (P10) mice were utilized to model HIE. A total of 110 mice (15 sham and 40 HIE mice for each sex) were used in this study, including 13 mice that were excluded from further assessments because of either death after HIE or failure in ischemia induction.

### Neonatal HIE Model

The Rice–Vannucci Model (RVM) of neonatal hypoxia–ischemia was modified to induce HIE in P10 mice^3,23^. Briefly, P10 pups were anesthetized with isoflurane (4% for induction and 1.5–2% for maintenance). A midline cervical incision was made and the right common carotid artery was exposed and double ligated with 6-0 silk suture thread. Sham mice underwent the same procedure except ligation of the right Common carotid artery (CCA). After surgery, the pups were returned to their dams for 2 hours. To induce hypoxia, the CCA-occluded mice were put in a chamber containing 10% oxygen and 90% nitrogen for 60 minutes. After that, the animals were placed on a temperature-controlled blanket for 20 min and then returned to their dams. Mice were sacrificed at 30 d after surgery for histological, cellular, and molecular analysis.

### Behavioral Testing

All behavioral tests were performed by a blinded investigator.

#### Neurological deficit scores

Neurological deficit scores (NDSs) were recorded at 1 h,1d, 2d, 3d of HIE. The scoring system used was as follows: 0, no deficit; 1, forelimb weakness and torso turning to the ipsilateral side when held by tail; 2, circling to affected side; 3, unable to bear weight on affected side; and 4, no spontaneous locomotor activity or barrel rolling as described previously^9^.

#### Seizure score

Seizure activity was scored according to a seizure rating scale as described previously^24,25^. Every 5 min in 1 h at 4 h and 24 h of HIE, the score corresponding to the highest level of seizure activity observed during that time period was recorded and summed to produce a total seizure score. Seizure behavior was scored as follows: 0 = normal behavior; 1 = immobility; 2 = rigid posture; 3 = repetitive scratching, circling, or head bobbing; 4 = forelimb clonus, rearing, and falling; 5 = mice that exhibited level four behaviors repeatedly; and 6 = severe tonic–clonic behavior.

#### Corner test

The corner test was performed to assess forelimb asymmetry at 30 days of HIE. The mouse was placed between two cardboard pieces (size of each: 30 × 20 × 1 cm). The two boards were gradually moved to close the mouse from both sides to encourage the mouse to enter into a corner of 30° with a small opening along the joint between the two boards. When the mouse entered the deep part of the corner, both sides of the vibrissae were stimulated together by the two boards. Animals with cerebral ischemia neglect the damaged side, and rear to the intact side (the right side) when the cardboard stimulates the vibrissae. The number and direction of rears are recorded for 20 trials, and the percentage of right turns was calculated. Only turns involving full rearing along either board were recorded^9^.

#### Wire hanging test

The wire hanging test was used to evaluate the motor function and deficit in stroke pups as described previously with slight modification^9,26^. A wire cage top (18 inch × 9 inch) with its edges taped off was used for this experiment. The mouse was placed on the center of the wire lid and the lid was slowly inverted and placed on top of the cage. The wire lid was 9 inches above the cage bedding. Latency to fall from the wire was recorded. The time out period was 90 seconds. The average performance for each session is presented as the average of the three trials.

#### Y-maze test

Spontaneous alternation using a Y-maze is a test for habituation and spatial working memory^27,28^. The symmetrical Y-maze consists of three white opaque plastic arms at a 120° angle from each other. After placing pups in the center, the animal is allowed to freely explore the three arms. Over the course of multiple arm entries, the subject should show a tendency to enter a less recently visited arm. The test consists of a single 5 min trial; spontaneous alternation (%) is defined as consecutive entries in three different arms (arm A, B, C), divided by the number of total alternations (total arm entries minus 2)^29^. Mice with less than 8 arm entries during the 5-min trial were excluded from the analysis. An entry occurs when all four limbs are within the arm.

### Assessment of Brain Tissue Atrophy by Cresyl Violet Staining

At day 30 (P40) after HIE, the animals were anesthetized with tribromoethanol (Avertin® intraperitoneal injection at a dose of 0.25mg/g body weight). Animals were perfused transcardially with ice cold 0.1 M sodium phosphate buffer (pH 7.4) followed by 4% paraformaldehyde (PFA); the brain was removed from the skull and post-fixed for 18 h in 4% PFA and subsequently placed in cryoprotectant solution (30% sucrose). The brain was cut into 30-μm free-floating coronal sections on a freezing microtome and every eighth slice was stained by cresyl violet ([CV] Sigma, St. Louis, MO, USA) for evaluation of ischemic damage^30^. Brain atrophy at 30d of HIE was computed by (1 − (ischemic hemisphere-ventricle-cavity) / (contralateral hemisphere-ventricle)) × 100 as previously described^31^.

### Flow Cytometry

Leukocytes from the brain tissue were prepared as previously described^3^. Briefly, animals (*n* = 6, HIE; *n* = 5, sham) were anesthetized with Avertin (2,2,2-Tribromoethanol, Sigma) and transcardially perfused with phosphate-buffered saline (PBS) for 5 min. The brain was then divided along the interhemispheric fissure into two hemispheres. Ipsilateral brains were placed in complete Roswell Park Memorial Institute (RPMI) 1640 (Lonza, Houston, TX) medium (Sigma), followed by mechanical and enzymatically digest with 150 µl collagenase/dispase (1 mg/ml) and 300 µl DNAse (10 mg/ml; both Roche Diagnostics, Risch-Rotkreuz, Switzerland) for 45 minutes at 37°C with mild agitation. The cell suspension was filtered through a 70 μm filter. Leukocytes were harvested from the interphase of a 70%/30% Percoll gradient. Cells were washed and blocked with mouse Fc Block (eBioscience, ThermoFisher Scientific, Waltham, MA, USA) prior to staining with primary antibody conjugated fluorophores: CD45-eF450 (# 48-0451-82, eBioscience), CD11b-AF700 (# 101222, Biolegend, San Diego, CA, USA), Ly6C-APC-eF780 (#47-5932-82, eBioscience), Ly6G-PE (#127608, Biolegend), MHCII-APC (#107614, Biolegend) and CD206-PE-cy5.5(#141720, Biolegend), and CX3CR1-PerCP-Cy5.5 (#149010, Biolegend). For each surface marker, 0.25 μg (1:100) of antibody was used to stain 1 × 106 cells. All the antibodies were commercially purchased from eBioscience. For live/dead discrimination, a fixable viability dye, carboxylic acid succinimidyl ester (CASE-AF350, Ivitrogen, Carlsbad, CA, USA, Sigma), was diluted at 1:300 from a working stock of 0.3 mg/ml. Cells were briefly fixed in 2% paraformaldehyde (PFA). Data were acquired on a CytoFLEX (Beckman Coulter, Brea, CA, USA) and analyzed using FlowJo (Treestar Inc., Ashland, OR, USA). No less than 100,000 events were recorded for each sample. Cell type-matched fluorescence minus one controls were used to determine the positivity of each antibody.

### Measurement of Serum Cytokines, Estradiol (E_2_), and Testosterone Levels by ELISA

Blood was taken at sacrifice from the right ventricle with heparinized syringes and centrifuged at 8000 rpm for 10 min at 4°C to yield serum. Serum was stored at −80°C until use. Enzyme-linked immunosorbent assays (ELISA) were performed using kits for testosterone (# ADI-900-065, Enzo Life Sciences, Inc., Farmingdale, NY, USA) and 17β-estradiol (# ES180S-100, Enzo Life Sciences) following the manufacturers protocol. Serum cytokines (tumor necrosis factor (TNF)-α, interleukin (IL)-10, IL-4) were measured by multiplex (Bio-Plex Pro™ Mouse Cytokine 8-plex Assay #M60000007A, BioRad, Hercules, CA, USA).

### BrdU Injection and Immunohistochemistry

After 7d of HIE, 5-Bromo-2′-deoxyuridine (BrdU; Sigma) was injected intra-peritoneally (50 mg/kg) to the animals for 5 consecutive days. Animals were sacrificed at 30 days of HIE and brain samples were prepared as described in CV staining. A cryostat was used to prepare free-floating brain slices from all mice (40 µm thick). For detection of BrdU incorporation, the brain slices were incubated in 50% formamide/2× standard sodium citrate for 2 hours at 65°C, incubated in 2 N HCl for 30 minutes at 37°C, rinsed in 100 mM boric acid (pH 8.5) for 10 minutes at room temperature, and washed with PBS (pH 7.4). The slices were then blocked in 0.1 M tris-buffered saline (TBS) with 0.3% Triton X-100 (Sigma, ThermoFisher Scientific, Waltham, MA, USA) and 10% donkey serum for 2 hours. For multiple antibody staining, coronal sections were incubated with following primary antibodies: rat anti-BrdU (BU1/75, 1:100, Novus Biologicals, Littleton, CO, USA), mouse anti-neuronal nuclei (NeuN; MAB377, 1: 1000, EMD Millipore, Darmstadt, Germany). After being washed in PBS, the sections were incubated with the indicated secondary antibodies for 1 h. For detection of microglia and astrocytes, brain slices were mounted onto gelatin-coated slides and allowed to air-dry. The sections were then blocked in 0.1 M PBS with 0.25% Triton X-100 (Sigma) and 10% donkey serum for 2 hours and incubated overnight at 4°C with the following primary antibodies: rabbit anti-Iba1 (#019-19741, 1:300, Wako, Osaka, Japan) and mouse anti-GFAP conjugated with Cy3 (#C9205;1:1000, Sigma). The following secondary antibodies were used: donkey anti-rabbit IgG Alexa Fluor 488 conjugate (A21206; 1: 500, Invitrogen), donkey anti-rabbit IgG Alexa Fluor 488 conjugate (A21207; 1: 500, Invitrogen) and donkey anti-rat IgG Alexa Fluor 594 conjugate (A11011; 1: 500, Invitrogen). The nuclei were stained with 4’,6-diamidino-2-phenylindole (DAPI; S36939, Invitrogen).

### Statistics

Investigators were blinded to mouse sex for stroke surgery, behavioral testing, infarct, and inflammation analysis. Mice with abnormal body weights and behavior (e.g. body shaking, limpness) before surgery were excluded. Data from individual experiments were presented as mean ± standard deviation (SD) and analyzed with a Student’s *t* test (atrophy volumes, hormone levels, and behavior tests) or two-way analysis of variance (cytokines and FC data). *P* < 0.05 was considered statistically significant. All analyses were done by using GraphPad Prism software (San Diego, CA, USA). The ordinal data of NDSs were analyzed with Mann–Whitney *U* test.

## Results

### Chronic HIE Outcomes in Male and Female Pups

**Figure 1.**
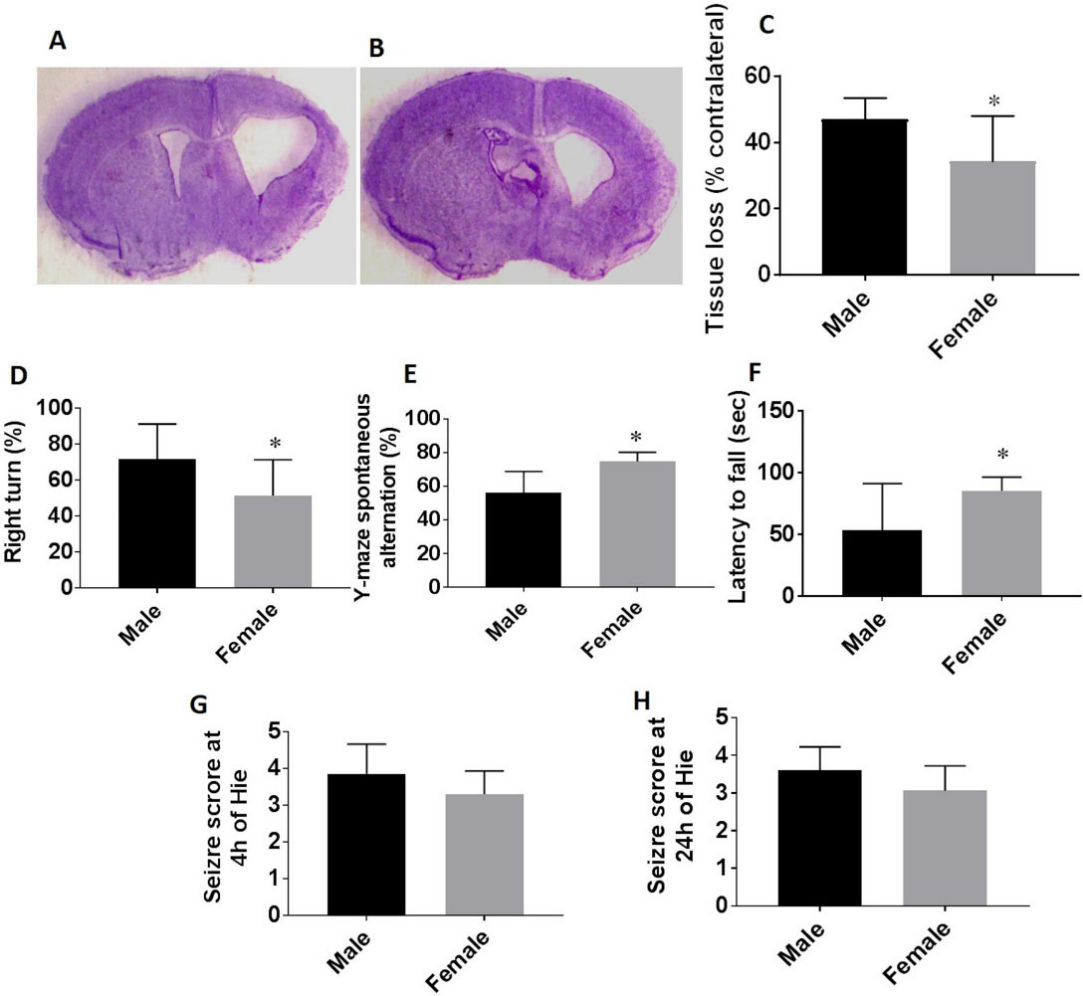
Chronic HIE outcomes at 30 days. (a–b) Representative images of brain slices stained with cresyl violet (CV) 30 days after HIE. Note that the cavitation and atrophy can be seen in both male and female brains. (c) Quantification of brain tissue loss. Functional outcomes were evaluated by the corner test, Y-maze, and wire hanging test. The percent of right turns (d), spontaneous alteration (e), and latency to fall (f) were significantly different in females (*p*< 0.05) than males measured in the corner test, Y-maze, and wire hanging test respectively. Seizure scores were not significantly different (*p* < 0.05) between male and female groups either at 4 h or 24 h after HIE (g, h). *N* = 9–10/group; *p < 0.05. HIE: hypoxic–ischemic encephalopathy

We examined outcomes at 30 days after HIE; the hypoxic–ischemic infarct becomes less visible at the chronic stage and the ischemic brains exhibit either cavitation or atrophy due to the tissue loss (Figure 1(a) and (b)). Quantitative data showed male animals had significantly more tissue loss than females (Figure 1(c)). We also performed a battery of behavioral assessment in HIE mice at the chronic stage to evaluate sensorimotor and cognitive deficits. In the corner test, the percent of right turn in male HIE pups was significantly higher than the female pups (Figure 1(d)). In the Y-maze, spontaneous alteration, which measures the willingness of rodents to explore new environments, was found to increase in female vs. male pups (Figure 1(e)), and the numbers of total arm entries in the Y-maze were not significantly different between groups (data not shown). Consistently, in the wire hanging test, female pups exhibited significantly longer latency to fall from the wire than their male counterparts (Figure 1(f)). No significant difference was seen in seizure scores (Figure 1(g) and (h)), and there was no sex difference in the mortality rate (20–25%).

### CD206 was Differentially Expressed on Male vs. Female Ischemic Microglia

**Figure 2.**
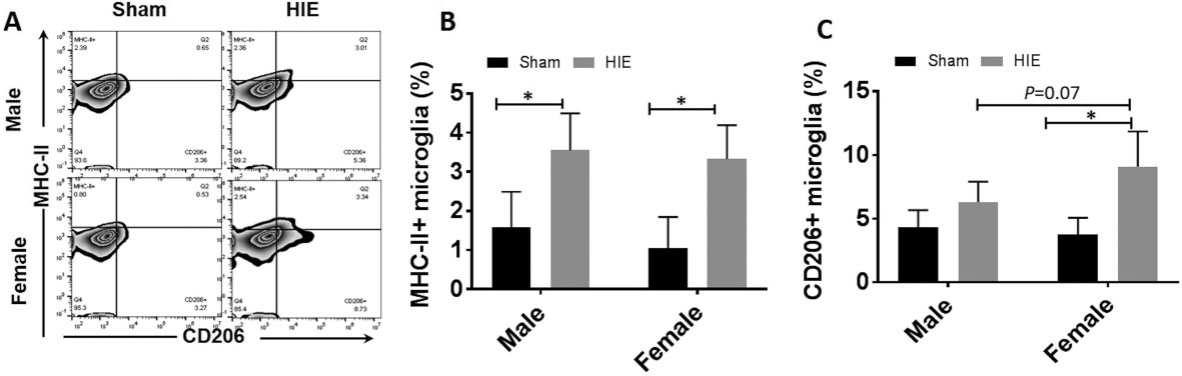
Microglial activation at 30 days after HIE. (a) Representative FC plot for MHC II^+^ microglia (up, left quadrant) and CD206^+^ microglia (down, right quadrant). Quantification of MHC II^+^ microglia (b) and CD206^+^ microglia percentage (c) of total gated microglia at 30 days after HIE. *n* = 6, HIE group; *n* = 5, sham group; *p < 0.05. HIE: hypoxic–ischemic encephalopathy; MHC: major histocompatibility complex

Microglia can be activated towards M1 or M2 polarization after an ischemic insult^9,32^. Our previous study has shown male pups had more MHC II^+^ microglia in acute HIE brains than females, suggesting a more robust pro-inflammatory microglial response in males^3^. To determine the state of microglial activation at the chronic stage, we performed FC to examine MHC II and CD206 expression on microglia as MHC II and CD206 are well-established markers for M1 and M2-like activation respectively^33,34^. We gated microglia as CD45^low^CD11b^+^, and then quantified the percentage of MHC II^+^ or CD206^+^ microglia. The gating strategy is shown in supplementary Figure 1. Our data revealed that both male and female mice had significantly more MHC II^+^ microglia in HIE vs. sham group. Although no sex difference was found in the percentage of MHC II^+^ microglia (Figure 2(a) and (b)), more CD206^+^ microglia was seen in female vs. male HIE group; in addition, only females (but not males) had significantly more CD206^+^ microglia in HIE vs. sham group (Figure 2(c)).

### CX3CR1 Expression in Microglia was Higher in Female vs. Male Ischemic Brains

**Figure 3.**
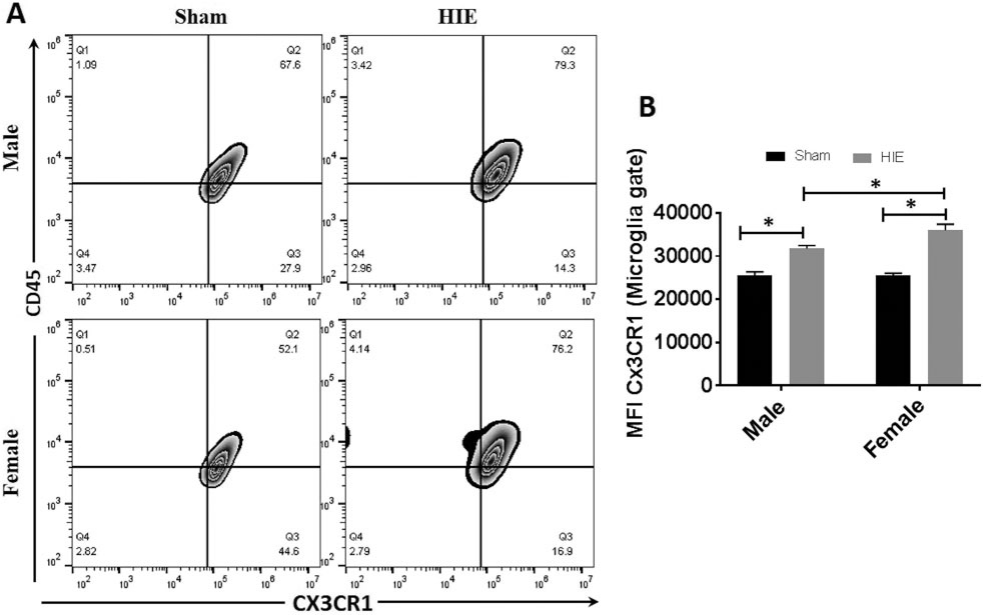
CX3CR1 expression in microglia at 30 days after HIE. (a) Representative FC plot for CX3CR1^+^ microglia (up, right quadrant). (b) Quantification of CX3CR1^+^ microglia of total gated microglia. *n* = 6, HIE group; *n* = 5, sham group; *p < 0.05. HIE: hypoxic–ischemic encephalopathy; CX3CR1: CX3C chemokine receptor 1; FC: flow cytometry.

CX3CR1 signaling has been shown to protect neurons by regulating microglial activation and migration^35,36^. Recent studies found sex differences exist in microglial CX3CR1 signaling in a mouse obesity model^19^. We also examined CX3CR1 expression with FC and quantified mean fluorescent intensity (MFI) of CX3CR1 on microglia in both male and female ischemic brains. At 30 days after HIE, the MFI of CX3CR1 in microglia significantly increased in both male and female mice compared with sham groups (Figure 3(a)). Interestingly, the MFI of CX3CR1 was significantly higher in female vs. male microglia after HIE. (Figure 3(b)).

### More Infiltration of Peripheral Leukocytes in Male vs. Female Ischemic Brains

**Figure 4.**
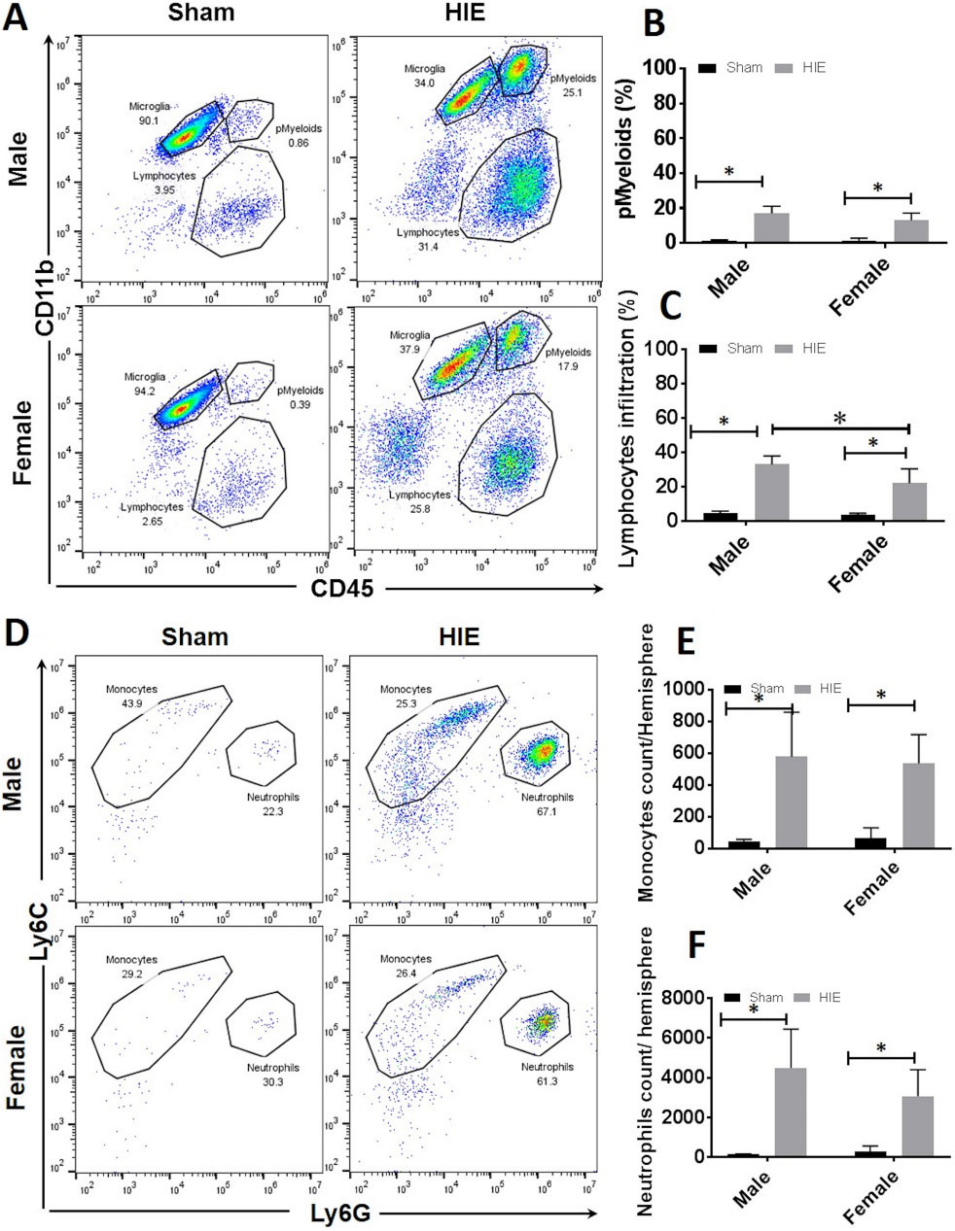
Infiltration of peripheral leukocytes in the brain at 30 days of HIE. (a) Representative FC plot showing microglia were identified as CD45^low^CD11b^+^ and infiltrating peripheral myeloid cells (pMyeloids) and infiltrating lymphocytes were identified as CD45^high^CD11b^+^ and CD45^low^CD11b^-^ respectively. (b, c) Quantification of % peripheral myeloid cells (b) and lymphocytes in total infiltrating leukocytes (c). (d) Representative FC plot showing monocytes were gated as CD45^high^CD11b^+^Ly6C^+^Ly6G^−^ and neutrophils as CD45^high^CD11b^+^Ly6G^+^. (e, f) Absolute counts of monocytes (e) and neutrophils (f) in HIE brain. *n* = 6, HIE group; *n* = 5, sham group; *p < 0.05. HIE: hypoxic–ischemic encephalopathy; FC: flow cytometry.

Inflammatory responses involve activation of resident immune cells (microglia) and infiltration of peripheral leukocytes in the ischemic brain. To evaluate the response of peripheral immune cells to chronic HIE, we quantified infiltrating leukocytes with FC. Total peripheral myeloid cells were gated as CD45^high^CD11b^+^, and lymphocytes as CD45^high^CD11b^−^, monocytes as CD45^high^CD11b^+^Ly6C^+^Ly6G^−^, and neutrophils as CD45^high^CD11b^+^Ly6G^+^ (Figure 4(a)). Quantitative data showed there were more peripheral leukocytes of each category in HIE vs. sham brains of both sexes, suggesting peripheral immune cells actively participate in the chronic inflammation of HIE. Additionally, there was significantly less lymphocyte infiltration in female vs. male brains (Figure 4(c)), and no sex difference was seen in other leukocytes infiltration (Figure 4(d–f)).

### Serum Cytokine Levels Exhibited Sex Differences at 30 Days After HIE

**Figure 5.**
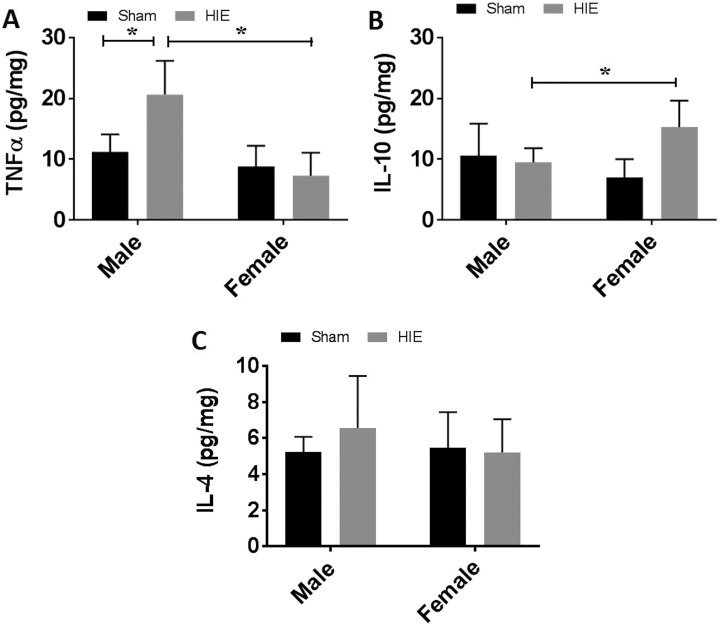
Cytokine levels in serum. (a) TNF-α levels were significantly higher in males than in females at 30 days after HIE. (b) Females had significantly higher IL-10 levels males. No sex difference was observed in IL-4 levels (c). *n* = 6, HIE group; *n* = 5, sham group; *p < 0.05. HIE: hypoxic–ischemic encephalopathy; IL: interleukin; TNF: tumor necrosis factor.

Since we found sex differences in immune cell infiltration (i.e. microglia and lymphocytes), next we wanted to know whether cytokine levels are also different between males and females after HIE. We examined serum levels of pro-inflammatory cytokine TNF-α and anti-inflammatory cytokines IL-10 and IL-4 with ELISA. Interestingly, we found the level of TNF-α was significantly lower in female pups compared with males (Figure 5(a)). In contrast, females had significantly higher level of IL-10 than their male counterparts (Figure 5(b)). No sex difference was observed in IL-4 cytokine levels (Figure 5(c)).

### More Neurogenesis in Female vs. Male Chronic HIE Brains

**Figure 6.**
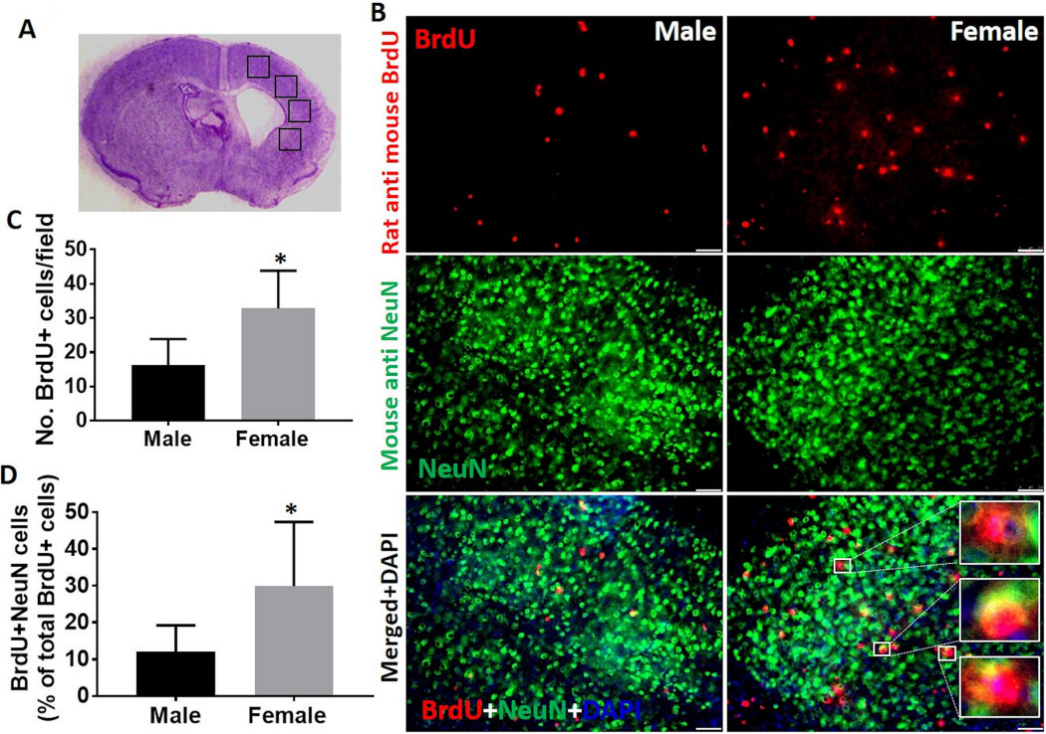
Neurogenesis at 30 days of HIE. (a) Image analysis were done in four ipsilateral cortical regions (black boxes) at the inner boundary zone of the atrophy. (b) Representative IHC images of BrdU, NeuN, and DAPI staining. Scale bar, 50 µm; 20× magnification. Inserts indicate 100× co-labeled cells. (c, d) Quantification of BrdU+ cells (c) and BrdU+NeuN double positive cells (d). *P < 0.05, *n* = 6/group. BrdU: 5-bromo-20-deoxyuridine; DAPI: 4’,6-diamidino-2-phenylindole; HIE: hypoxic–ischemic encephalopathy; NeuN: neuronal nuclei.

To evaluate whether neurogenesis was different between male and female HIE brains at the chronic stage, we performed immunohistochemistry to examine BrdU positive cells in the peri-infarct area (Figure 6(a)). The total BrdU positive cell number was significantly higher in female vs. male HIE brains (Figure 6(b) and (c)). More importantly, female HIE brains had significantly more BrdU and NeuN double positive cells than male brains (Figure 6(b) and (d)), indicating neurogenesis is more active in response to the HIE insult in female brains. We also examined microglia and astrocytes with immunohistochemistry (IHC), and did not find any sex difference in microglia or astrocyte morphology at 30d of HIE (Supplementary Figures 2 and 3).

### Changes in Gonadal Hormone Levels After HIE

**Figure 7.**
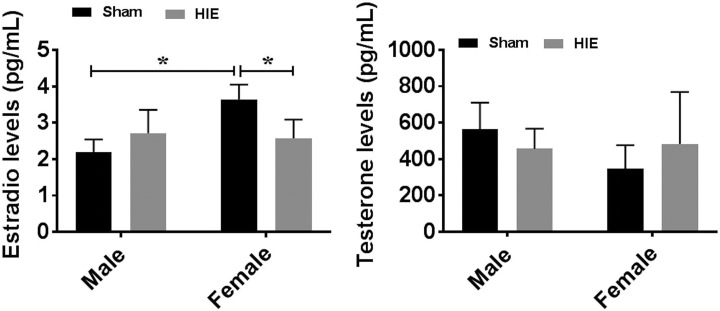
Gonadal hormone levels by ELISA at 30 days of HIE. (a) E2 levels were significantly decreased in females after HIE; no significant difference was seen between male and female HIE groups. (b) Testosterone levels were not significantly different between males and females. *n* = 6, HIE group; *n* = 5, sham group; *p < 0.05. ELISA: enzyme-linked immunosorbent assay; HIE: hypoxic–ischemic encephalopathy.

To investigate whether the sex differences in HIE outcomes and immune responses were related to gonadal hormones, we measured serum levels of testosterone and E_2_ 30 days after HIE. As expected, female mice had significantly higher levels of E_2_ levels than males in sham groups. Surprisingly, HIE induced a significant decrease in E_2_ levels in females, so much so that both male and female mice had equivalent E_2_ levels at the chronic stage of HIE (Figure 7(a)). However, no significant differences in testosterone (Figure 7(b)) levels were seen between males and females at either sham or stroke group. The hormone data suggested factors other than hormones are responsible for the differing HIE outcomes in male vs. female mice.

## Discussion

Previous studies have elucidated sex differences in the acute phase of neonatal HIE^3^. In the present study, we utilized a widely accepted HIE model (RVM) to study sex differences in chronic neonatal HIE outcomes and revealed several important new findings. Firstly, sex differences exist in chronic HIE either in morphology or in behavior deficits, and sex hormones may not be determinants in inducing the sex difference as males and females have equivalent circulating hormone levels after chronic HIE. Secondly, female HIE microglia exhibit a more robust anti-inflammatory response (M2) than male microglia, indicated by more highly expressed CD206. Interestingly, the higher level of female M2-like microglial activation was corresponding to a marked increase in CX3CR1 expression. Thirdly, peripheral immune responses are still active in the chronic stage of HIE, and are sex specific in the lymphocytic response and serum cytokine levels. Lastly, more new-born cells (BrdU^+^ cells) were seen in female vs. male HIE brains, suggesting neurogenesis was more highly induced in female brains where a stronger recovery was mounted after HIE injury. To our knowledge, this is the first study that examined the sex differences in central and peripheral immune responses to chronic HIE, and highlighted the mechanisms of neuroprotection in female HIE brains.

Brain ischemia is a powerful stimulus that triggers a series of events that cause resident microglia to become activated and develop macrophage-like capabilities^7^. Previous research demonstrated that microglial activation has two distinct phenotypes: a pro-inflammatory state (classical activation, M1) and an anti-inflammatory state (alternative activation, M2)^37^. Different microglial phenotypes are well known to exert distinct effects on stroke pathophysiology and brain repair. Specifically, the ‘classical activation’ M1 microglia release destructive pro-inflammatory mediators and exacerbate brain damage. In contrast, the ‘alternative activation’ M2 phenotype is essential for tissue preservation and brain repair because M2 cells resolve local inflammation, clear cell debris, and provide trophic factors^38^. The lack of necessary endogenous signals for M2 induction is known to worsen outcomes after cerebral ischemia^39^. The present study revealed that the MHC II^+^ microglia, a widely used marker of M1 microglial activation, were significantly upregulated in HIE vs. sham mice of both sexes after 30 days of stroke (Figure 2(b)), suggesting the pro-inflammatory response can persist in neonatal ischemic brains and therefore may be an attractive target for therapeutic intervention. Induction of proteins in microglia with key functions in antigen presentation and inflammation (e.g. MHC II) seems to be confined to microglial subpopulation, and discrete synthesis of MHC II after HIE controls the activation of M1 microglia in both male and female mice^40^. CD206 is a classic M2 marker expressed by microglia/macrophages whose main function is to restrict the M1 microglia/macrophages polarization^9,10^. The present study further found that females had more CD206^+^ cells than males at 30 days of HIE (Figure 2(c)), indicating more anti-inflammatory microglia in females that keep ‘fighting’ and eventually outweigh the pro-inflammatory response to conduct tissue repair, which may contribute to the better outcomes in females.

Activation of the CX3CL1/CX3CR1 axis is neuroprotective in multiple neuroinflammatory diseases, including ischemic stroke. Deleting CX3CR1 exacerbated microglial activation and elevated levels of inflammatory cytokines^41^. Similarly, systemic Lipopolysaccharide challenge increased production of IL-1β by microglia isolated from the brain of Cx3cr1-/- mice^42^. Moreover, CX3CL1 level decreased in the brain of aged mice possibly due to neuronal loss which further increase the expression of pro-inflammatory cytokines such as IL-1β with LPS challenge in comparison with young mice^43^. Recent study found that sex differences in microglial activation exist in the modulation of energy homeostasis and identified CX3CR1 signaling as a potential therapeutic target for the treatment of metabolic diseases in mice such as obesity^19^. We also found a sex difference in CX3CR1 signaling in the chronic HIE (i.e. female microglia had higher expression of CX3CR1 than male microglia) which is corresponding to the more robust anti-inflammatory response in females. It is likely that female microglia are more primed towards M2 activation due to increased expression of microglia CX3CR1 (Figure 3(b)); as a result, females may benefit more than males from the protective signaling.

In the present study, we revealed a sex difference in the chronic HIE inflammation evidenced not only by microglial activation but also by peripheral immune responses (Figures 2, 4, 5). There were significantly more lymphocytes infiltrating in male vs. female brains after 30 days of HIE, although the peripheral total myeloid cells, monocytes, and neutrophils did not differ by sex significantly, suggesting lymphocytic response is a main driving force to sex specifically induce peripheral immune response to chronic HIE^44^. The lower level of lymphocyte infiltration in female mice brains might contribute to the better chronic HIE outcomes and suppression of lymphocyte infiltration might be more effective in treating male HIE. TNF-α is a major pro-inflammatory mediator whose level increases after HIE^45^. High-levels of TNF-α can lead to local inflammatory responses causing damage to body tissues and organs. Previous studies have shown that severe brain damage stimulates the release of TNF-α and other inflammatory molecules into systemic circulation. In the present study, we observed a higher serum level of TNF-α in the HIE vs. sham mice only in male but not in female groups. More importantly, males have higher levels of TNF-α than females 30 days after HIE, suggesting a more robust pro-inflammatory response in male vs. female HIE mice^3^. IL-10 is an anti-inflammatory cytokine, and can induce an ‘alternatively activated’ M2 microglia/macrophage phenotype that possesses neuroprotective properties after cerebral ischemia^46^. Interestingly our data showed sex difference also exists in IL-10 serum levels after HIE (i.e. female mice had significantly higher levels IL-10 than males) suggesting an upregulated, chronic anti-inflammatory response in females.

The time point examined in the present study (i.e. 30 days after HIE) was an age equivalent to that of human adolescence^47^. Therefore, circulating hormones may contribute to the sex differences in HIE outcomes and inflammatory responses. Surprisingly, our data showed both testosterone and E_2_ levels were not significantly different between male and female mice after HIE, although there was a baseline sex difference in E_2_ levels (Figure 7). In adult stroke models, the ‘male sensitive’ stroke phenotype has been attributed to E_2_’s protection^48,49^ that is however, unlikely to contribute in our chronic HIE model, as E_2_ level in females was decreased to that of males after HIE. Why HIE injury decreases E_2_ levels is not clear. One possible reason might be that hypoxia causes the pituitary injury and consequently leads to the reduction of gonadotropin^50^.

In summary, the present study demonstrated sex differences in long-term HIE outcomes and immune responses. Although the direct mechanisms underlying the sex difference are still elusive with the current data, we revealed significant cross-talks between the sex-specific outcomes and chronic inflammatory responses, as well as the different neurogenesis in male and female HIE mice. Microglial activation continues to play a critical role in mediating HIE injury at the chronic stage, with a favorable effect in females; whereas lymphocytes are the main contributor of the peripheral compartment to the sex difference. Circulating hormones, however, are not contributing factors to this sexual dimorphism in the chronic HIE.

## Supplementary Material

Supplementary material

Supplementary material

Supplementary material
