# Supplementary figures and images for: Inflammatory Responses are Sex Specific in Chronic Hypoxic–Ischemic Encephalopathy
Source: Cell Transplant. 2018 Apr 25;27(9):1328–39. doi: 10.1177/0963689718766362 (PMC6168990; doi:10.1177/0963689718766362)

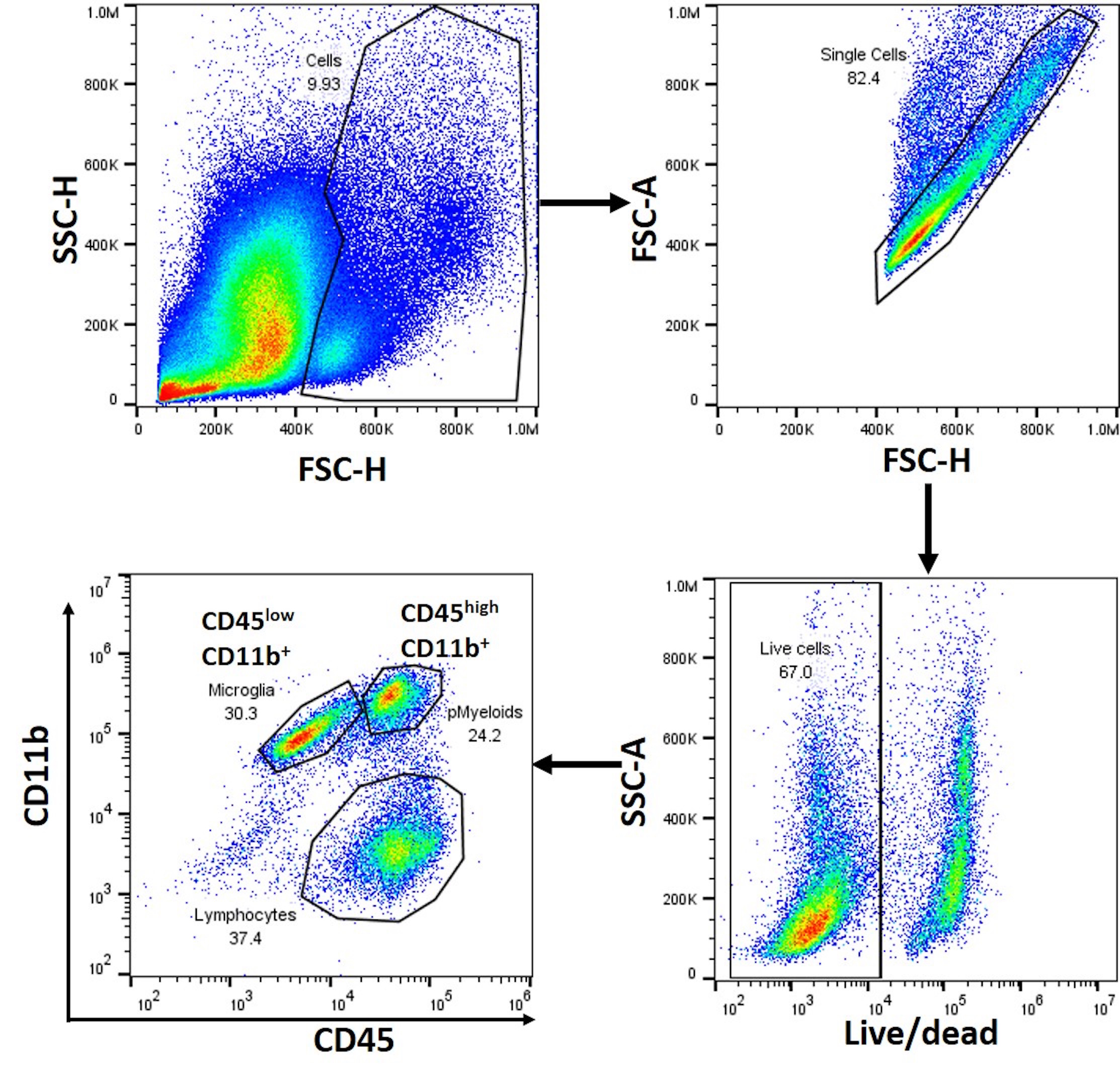

Supplement: Supplementary material [file 766362_Sup.figure1.jpg]

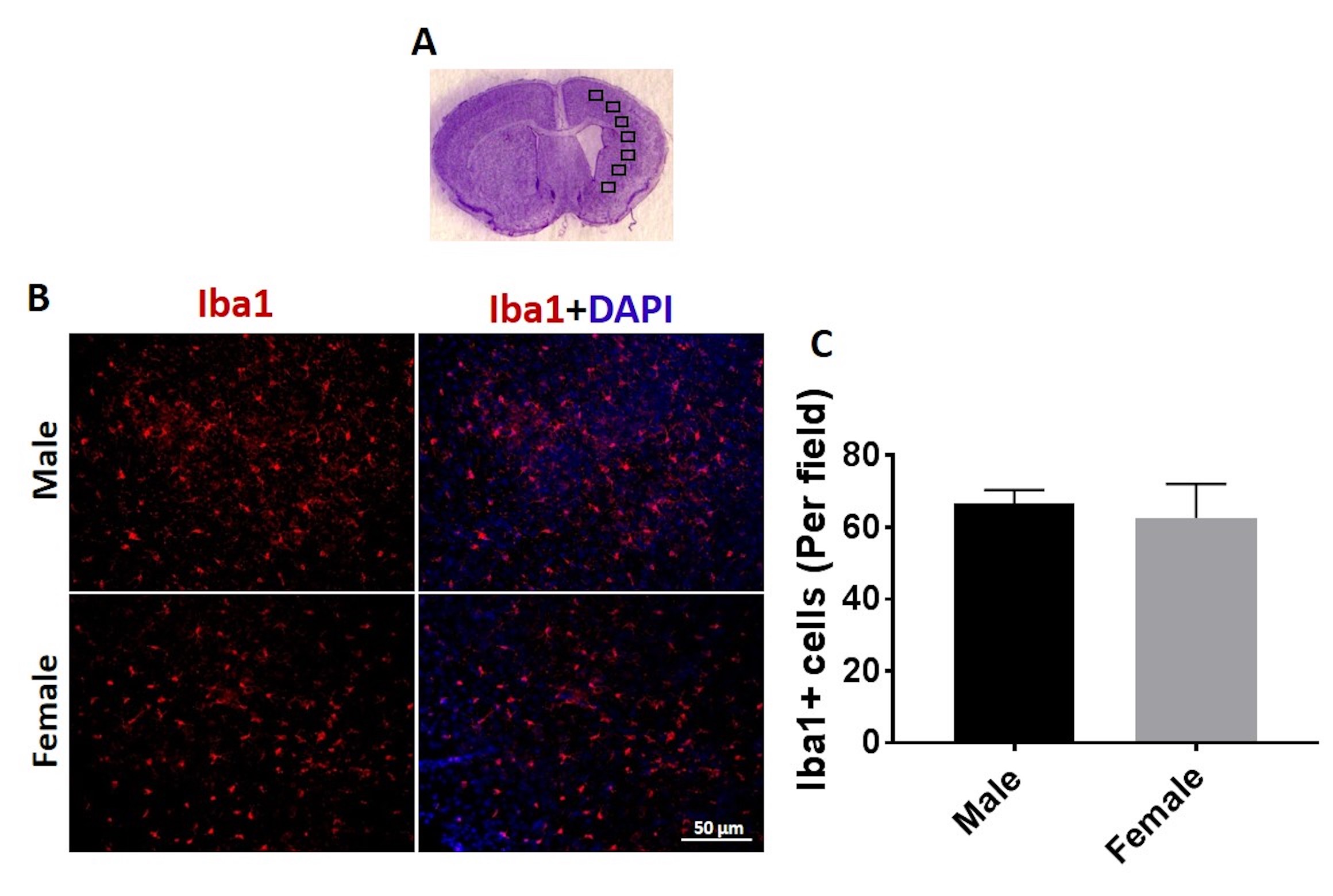

Supplement: Supplementary material [file 766362_Sup.figure2.jpg]

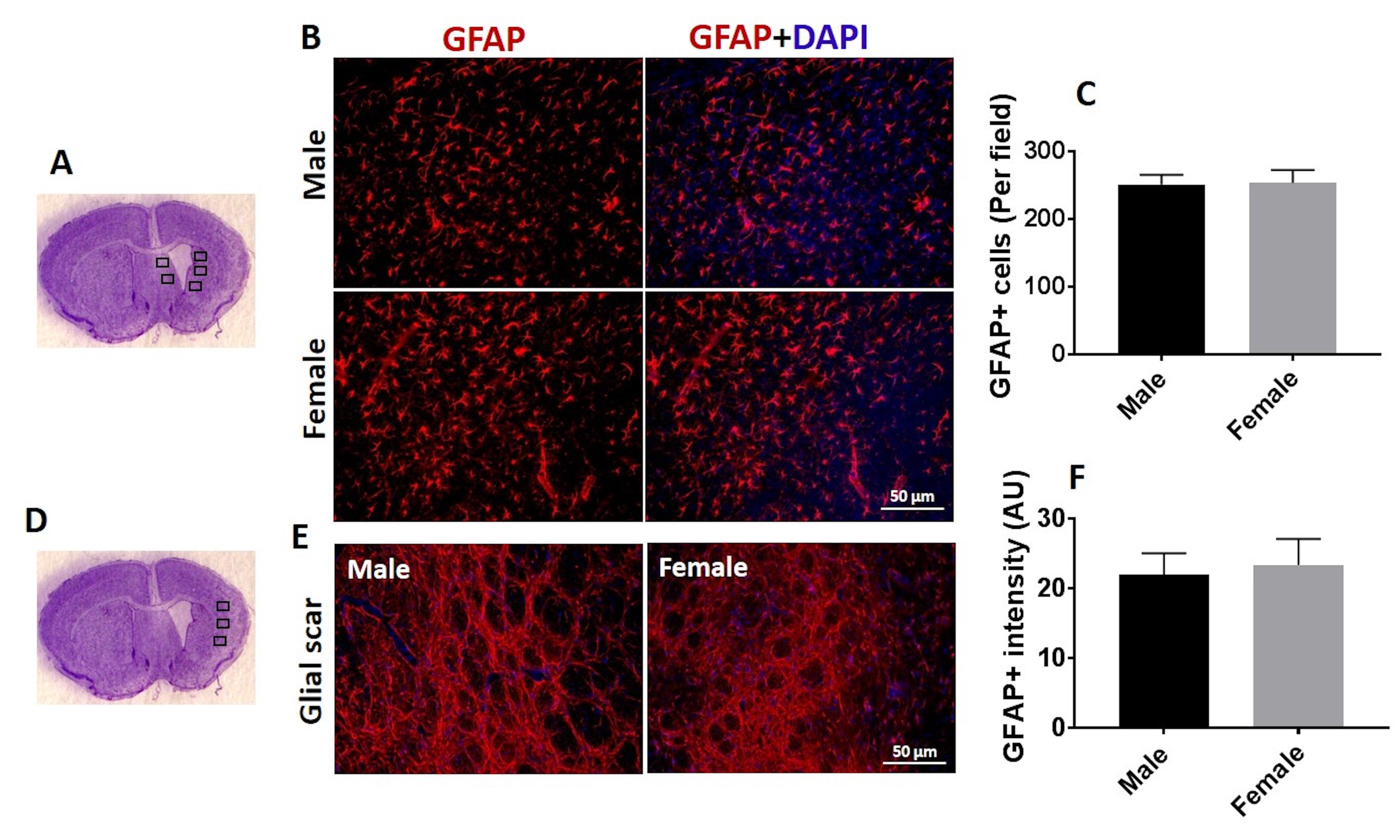

Supplement: Supplementary material [file 766362_Sup.figure3.jpg]
